# Adsorption behavior of trace elements of ^90^Sr on MnO_2_–ZrO_2_ loaded with polyacrylonitrile polymer from aqueous solutions

**DOI:** 10.1038/s41598-023-48010-x

**Published:** 2023-11-22

**Authors:** Neda Akbari, Seyed Javad Ahmadi, Akram Pourmatin, Mehran Heydari, Zahra Shiri-Yekta

**Affiliations:** Nuclear Fuel Cycle Research School, Nuclear Science and Technology Research Institute, Tehran, Iran

**Keywords:** Analytical chemistry, Nuclear chemistry

## Abstract

A MnO_2_–ZrO_2_-polyacrylonitrile (MnO_2_–ZrO_2_-PAN) composite ion exchanger was produced and its properties were examined by Fourier-transformed infrared spectroscopy, scanning electron microscopy, The BET (Brunauer, Emmett and Teller) surface area, X-Ray diffraction analysis and thermogravimetric analysis. The adsorption of Strontium (Sr) from solutions by MnO_2_–ZrO_2_-PAN composite was studied thru batch experiments. The distribution Coefficient of Sr (II) on the composite sorbent was investigated against pH, interaction time, and primary concentration ion. To study the kinetics of adsorption, Pseudo-first-order and Pseudo second-order adsorption kinetics were studied and the results revealed that adsorption kinetics better fit to the pseudo-second-order model. Three iso-temperature models, Langmuir, Freundlich, and Temkin were applied to fit the experimental results. Among those models, Langmuir revealed the most suitable one with minimum deviation. The created composite exhibited strong compatibility to the elimination of Y (III), Ni (II), Pb (II), and Co (II) from radioactive waste streams. On the other, it is evident from the data that the quantifiable extraction of Sr (II) ions from Zr (IV), Mo (VI), and La (III) is feasible. MnO_2_–ZrO_2_ Loaded with (PAN) Polymer was figured out to have high ion exchange capacity and thermal stability and selectivity for strontium.

## Introduction

Decontamination and treatment of nuclear sites with waste streams and elimination of radioactive contaminants are one of the most important problems in nuclear power.

Sr (II) is the high plentiful radioactive nuclides in fission products which are regularly or coincidentally liberated that its separation from liquid nuclear wastes has been attracted the attention of the relevant nuclear industries. It has a somewhat long half-life of around thirty years and is known as a harmful component for humans and life. A similar metabolic behavior to that of calcium leads to radionuclide accumulation in bone tissues. Once ingested, it can substitute calcium in the bone structure of living organisms and acts as a long-term source of the irradiation of bone marrows that makes strontium-90 one of the most dangerous radionuclides to human health. Therefore, the removal of strontium among all other contaminants from nuclear facility-related wastewaters is of great interest because of its relatively long half-life and high radiological toxicity. Radioactive isotope 90Sr is an example of a contaminant that is difficult to separate from the aqueous medium using conventional methods^1,2^.

The capability of ion exchangers to withdraw trace ions from suspension and the concentration that can be attained by cleaning out with an appropriate solvent were utilized in the treatment and retrieval of metals from highly diluted mixtures. Ion exchangers were employed broadly in wastewater treatment in the metal plating treatment for instance, where useful metals are extracted at expenses not as much of traditional system, with considerably less treatment area for the process. Moreover, several ion exchangers like zeolites, sodium titanates, titanosilicates, hexacyanoferrates, acidic salts of multivalent metal, salt of heteropoly acids, and hydrous oxides were studied for the elimination of fission products^3–6^.

There have been numerous published papers that discussed the various methods for uptake of strontium-90^7–10^. Numerous researchers evaluated the adsorption of Sr (II) from waste resources thru different organic and inorganic ion exchangers and it was obtained that an inorganic ion exchanger has some benefits compared to the organic one, involving upper thermal stability, and consistency in acidic to alkaline mixtures, and high adaptability with the ultimate waste characteristics. Additionally, as a result of their characteristic regarding pure inorganic ion exchangers, composite ion exchangers has been newly employed for their superior selectivity for the adsorption of various metal ions, improved chemical and mechanical strength, minor solubility in solutions and more acceptable kinetics of exchange against pure inorganic ion exchangers^11^.

Ahmadi et al.^12^ examined a MnO_2_–ZrO_2_ ion exchanger for the extraction of Sr (II) ions from waste systems. This exchanger has a high exchange capacity and good selectivity compared to other ion exchangers and the amount of strontium adsorption for the resin synthesized in this work is higher compared to other reported adsorbents.

To intensify the adsorption capability and improve the physico-chemical characteristics of MnO_2_–ZrO_2_, it is essential to employ a polymer binder that has low cost and is accessible as a raw matter. Polyacrylonitrile was offered as a common binding polymer for almost any inorganic ion exchangers. Spherical beads formed by this method have advantages of classical organic ion exchangers as well as the selective parameter of inorganic ion-exchangers^13^. Numerous papers were written about the application of PAN as a binding polymer for various inorganic cation exchangers^14–17^.

In the current study, the synthesis of a novel adsorbent based on polyacrylonitrile was considered to have a high potential as an adsorbent due to its physico-chemical characteristics and removal of harmful elements such as strontium from waste.

In this work, organic and inorganic ion exchangers composite were produced and consumed for the removal of Sr (II) from the aqueous mixture. The impact of different operational factors for instance interaction time, pH and primary concentration cations is examined and the data attained are reviewed. Additionally, equilibrium iso-temperature models for (Sr) ions adsorption on the ion exchangers were evaluated by the adsorption records as well.

## Experimental

A typical aqueous solution of Sr (II) was produced by mixing Sr (NO_3_)_2_ in deionized water. Amorphous MnO_2_–ZrO_2_ composite was produced based on a described procedure^12^, in size (100 μm). PAN as a binding polymer was attained from Aldrich. All the other substances and chemicals consumed were achieved from Aldrich with analytical grade. The pH is calculated by a Schott pH-meter, model CG841; the (FT-IR) was examined by a Brucker Vector 22 spectrophotometer using KBr disks; The BET surface area and average pore size of the products were measured by N^2^ adsorption–desorption test (Quanta chrome measuring instrument), powder XRD patterns were obtained using a Rigaku Powder X-Ray diffractometer which is equipped with a cobalt tube, graphite monochromator and scintillation detector, TGA analysis was performed with a Rheometric scientific 1500 using a heating rate of 10 °C/min; electron micrographs were recorded with a (SEM) apparatus, Philips XL-30; and Varian liberty 150 XL inductively coupled plasma (ICP) was applied for analysis of Metal ions. A water bath shaker is occupied for all experimental tests at equilibrium state.

### Synthesis of MnO_2_–ZrO_2_

An aqueous MnO_2_–ZrO_2_ was made by blending 0.5 M ZrOCl_2_ mixture to 0.5 M Mn (NO_3_)_2_ mixture with a ratio 2:1 (Zr/Mn) in volume based. A mixture of Potassium hydroxide (1 M) was introduced in drops to the former mixture to create pH = 10. Subsequent solutions were agitated for an hour at 25 °C. The sediment remained overnight, filtered, and later eluted many times with water till the remains were nearly neutral, and desiccated at 50 °C^12^.

### Synthesis of MnO_2_–ZrO_2_-PAN composite

PAN was applied as a common binding polymer for several inorganic ion exchangers. The composite of ion exchangers (MnO_2_–ZrO_2_) with polyacrylonitrile (MnO_2_–ZrO_2_-PAN) was produced by dissolving the MnO_2_–ZrO_2_ powder in dimethyl sulfoxide (DMSO) which involved 4% (w/w) sodium dodecyl sulfate surfactant and stirred for 2.5 h and in other container polyacrylonitrile were blended with DMSO to make a sticky polymer solution. Afterward, the substances of the two containers were combined and agitated to attain a uniform solution of the composite dope. The attained composite mixture was introduced to the binary nozzle and sprayed in water that involved surfactant to create the composite with a spherical shape. The composite beads have been cleaned with deionized and dried overnight at 50 °C to eliminate the solvent.

### Batch studies

The ion exchanger capacity of MnO_2_–ZrO_2_-PAN composite for K^+^ in mixtures was examined thru batch experiment^18,19^ with 200 mg of solid and 20 ml of 0.05 M of the KCl solution in a water bath, Shaked and set at 25 ± 1 °C till equilibrium was reached. After the equilibrium state, the phases were isolated and evaluated. The capacity (meq/g) was calculated from:

1 $$ {\text{Capacity (meq/g)}} = C_{0} \cdot {\text{Z}} \cdot \frac{{{\text{\% Uptake}}}}{{{100}}} \cdot \frac{V}{m} $$

2 $$ {\text{\% Uptake}} = \frac{{{\text{(C}}_{{0}} - {\text{C}}_{{\text{e}}} {)}}}{{{\text{C}}_{{0}} }} \times {100} $$

where *C*_o_ denotes the primary concentration and *C*_e_ considered as an equilibrium concentration of ions (mg/L), V is the solution volume (ml), m is the exchanger weight (g) and Z is the adsorbed (MI) charge^20^. The ionic exchange capacity of the resultant MnO_2_–ZrO_2_-PAN was calculated to be 1.2 meq g^−1^.

A Distribution Coefficient is the concentration of a component in the ion exchanger (the stationary phase) divided by its concentration in the external solution (the mobile phase) in equilibrium. The distribution Coefficient, K_d_ (mL/g) demonstrated the selectivity of the composite and the highest processing capacity of the various examined cation. The amounts of K_d_ were defined in batch runs. A weighed quantity of exchanger (0.2 g) was agitated for five hours at 22 ± 1 °C in a polymeric container including 20 ml of 10 mg/L of (MI) mixture. The concentrations (mg/L) of the mixture prior to equilibrium and afterward were calculated by ICP method. The distribution Coefficient amounts were determined based on the following correlation:

3 $$ K_{d} \left( {{\text{mL}}/{\text{g}}} \right) = \left( {\frac{{C_{0} }}{{C_{e} }} - 1} \right) \times \frac{V}{m} $$

## Results

### Identification and characterization

**Figure 1:**
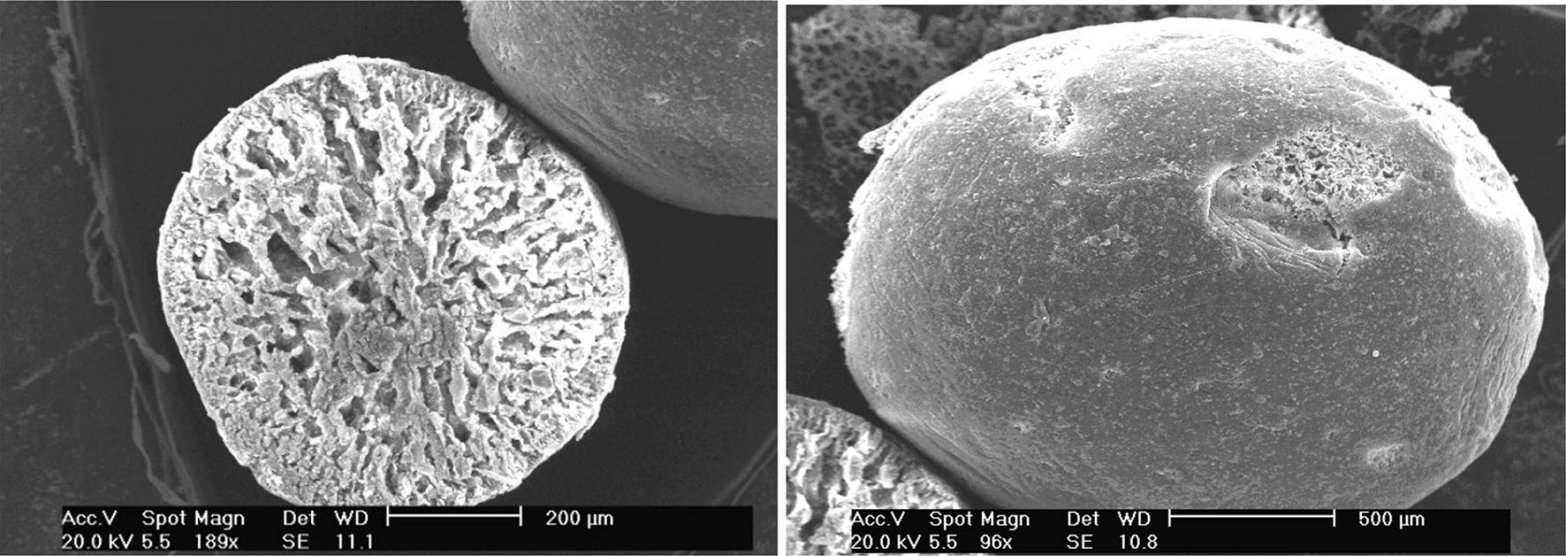
Scanning electron microscopic photograph of MnO_2_–ZrO_2_-PAN.

The results of the scanning electron microscopy of MnO_2_–ZrO_2_-PAN showed that the particles were heterogeneous (Fig. 1). A shell of MnO_2_–ZrO_2_-PAN with many cavities was detected; the porosity of the deep part of the particles was considerable than that of the near surface.

**Figure 2:**
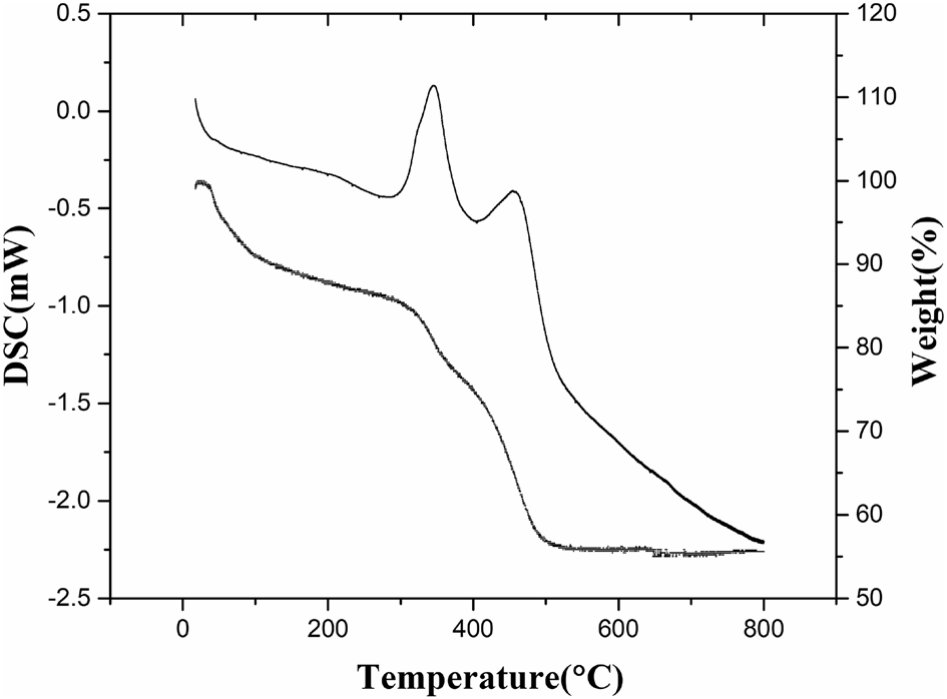
TGA–DSC curves of MnO_2_–ZrO_2_-PAN.

The TGA results related to MnO_2_–ZrO_2_-PAN are displayed in Fig. 2 which presents 4 decomposition stages. The first one looks obvious from TG data and occurred between 60 and 300 °C with a weight loss of 14.06% as a result of the removal of external moisture. The next step took place between 300 and 400 °C with a rapid and extreme heat release with the utmost of 350 °C related to a weight loss of 9.5%. The exothermic peak is attributed to the agglomeration of nitrile groups of PAN. Finally, the last peak found between 400 and 600 °C, with the utmost of 460 °C related to weight loss of 39.5% could be because of the full liberation of polymer chain fragments^15^. The major elements were analyzed by XRF and the existence of Zr and i elements was confirmed using XRF technique and the percentage of each oxide was determined. (Table 1).

**Table 1:** XRF Analysis of MnO_2_–ZrO_2_-PAN.

Analyte	Concentration
MnO_2_	47.5 Wt%
ZrO_2_	41.4 Wt%
Na_2_O	2.1 Wt%
Al_2_O_3_	1.1 Wt%
SO_3_	1.4 Wt%
Cl	3.8 Wt%
K_2_O	300 ppm
CaO	750 ppm
SC_2_O_3_	550 ppm
TiO_2_	350 ppm
V_2_O_5_	600 ppm
Fe_2_O_3_	0.3 Wt%
NiO	0.2 Wt%
As_2_O_3_	800 ppm
La_2_O_3_	0.2 Wt%

**Figure 3:**
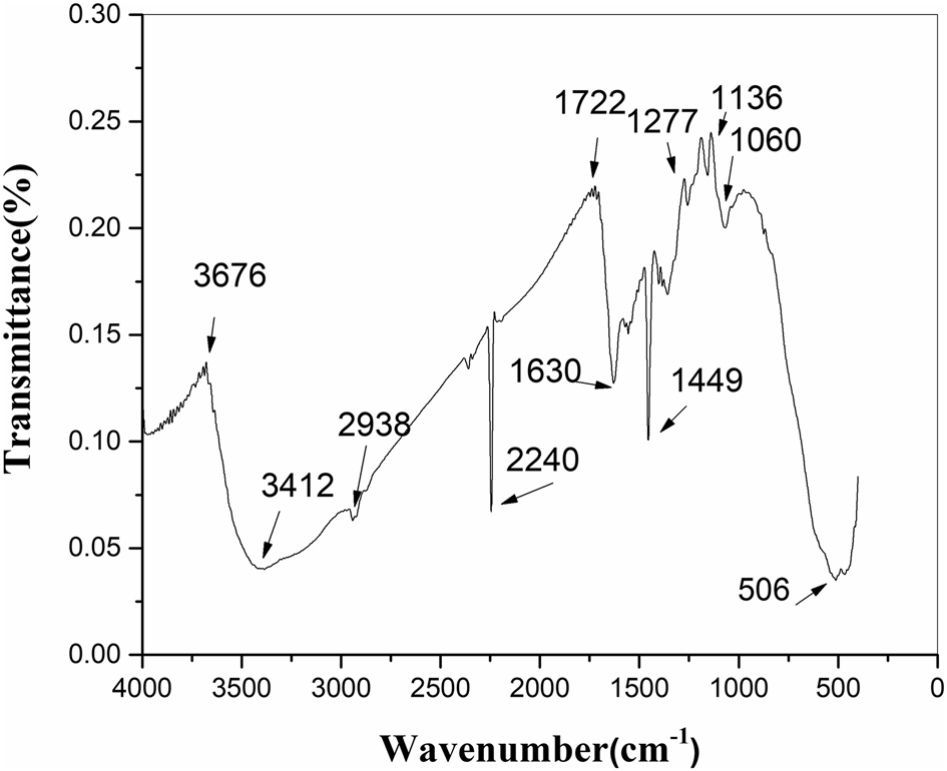
Infrared spectrum of MnO_2_–ZrO_2_-PAN.

**Figure 4:**
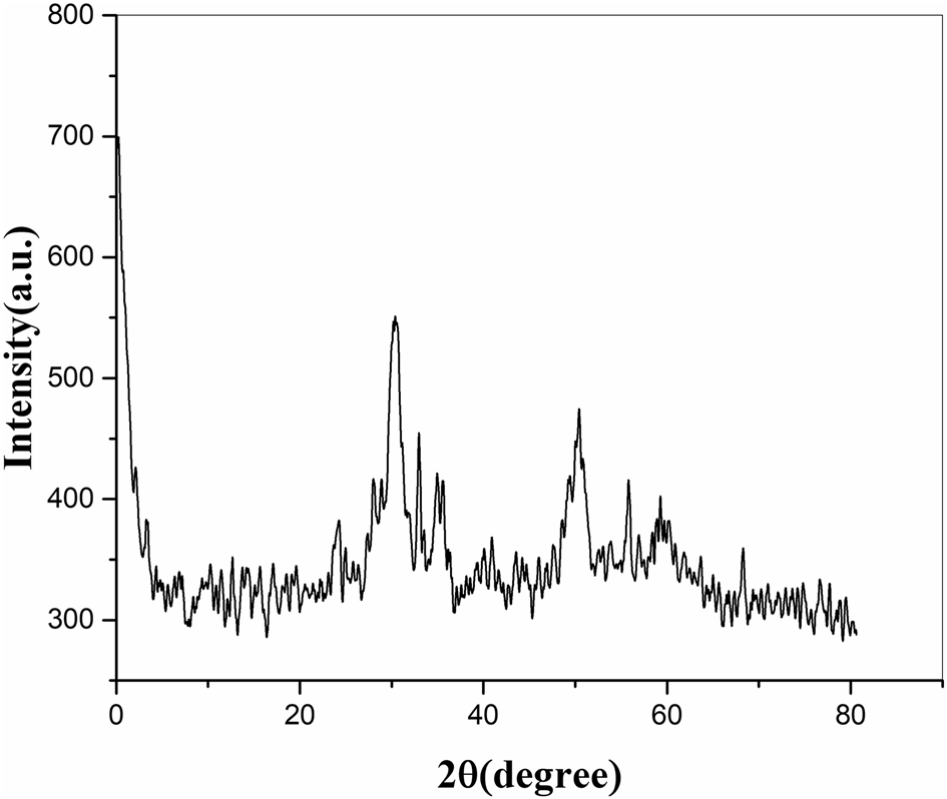
X-Ray diffraction analysis (XRD) of MnO_2_–ZrO_2_-PAN.

An image of FTIR spectrum in Fig. 3 indicates the wide band detected around 3000–3500 cm^−1^ that specifies the presence of the –OH group. The peaks at 1000 and 1070 cm^−1^ belong to the Metal-OH group of adsorbed water. The intense bands at 2250 cm^−1^ approved the stretching vibration of CN and 1460 cm^−1^ as a result of the CH bending of methylene groups of PAN, proving the existence of the organic moiety in intercalation compound spectra^15^. The results of BET analysis showed that the surface area was calculated 311 m^2^/g and the average pore diameter was considered 2.95 nm. Figure 4 shows XRD patterns of MnO_2_–ZrO_2_-PAN with PDF2 standard identifies that result was compatible with MnO_2_ (PDF# 01-0799) and ZrO_2_ (PDF# 013-0307). The XRD pattern for synthesized MnO_2_–ZrO_2_-PAN shows ZrO_2_ diffraction significant peaks that correspond to the monoclinic form. The peaks at 30°, 35°, 50° and 60° indicate that the crystallinity of ZrO_2_ component.

### Preliminary adsorption experiments

**Figure 5:**
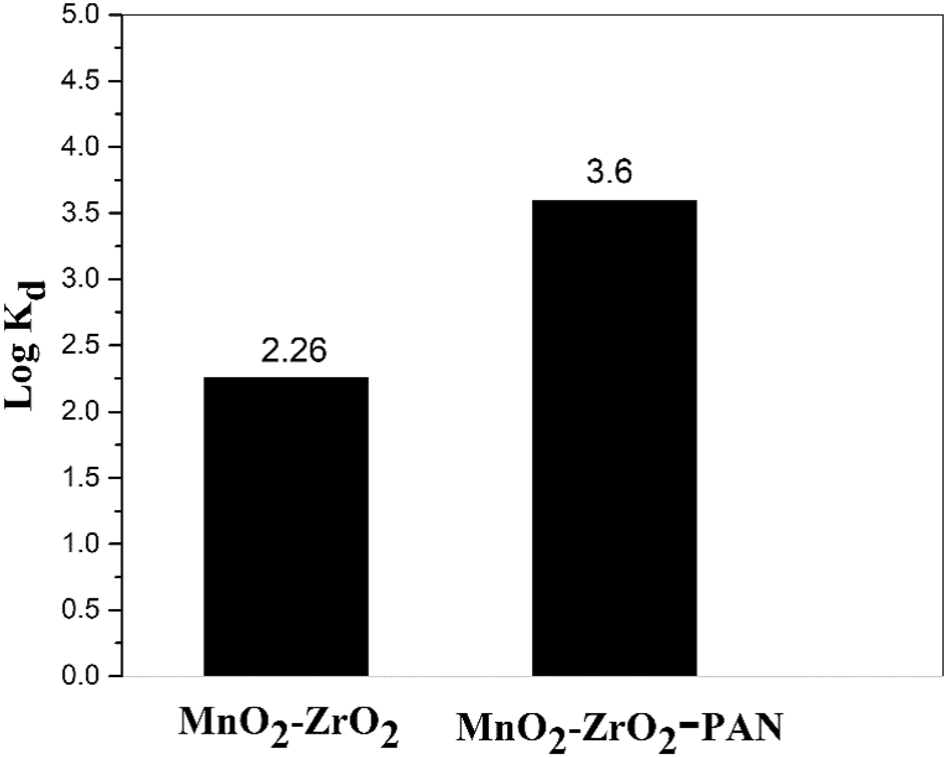
Distribution coefficient of strontium ions onto MnO_2_–ZrO_2_ and MnO_2_–ZrO_2_-PAN composites.

A series of adsorption experiments of (Sr) ions from solution into MnO_2_–ZrO_2_ and MnO_2_–ZrO_2_-PAN for comparison ability absorbers in experimental conditions: initial concentration of the Metal ions 50 mg/L; volume of aqueous phase 10; sorbent mass 0.10 g; aqueous phase pH 5.5; stirring time 60 min; temperature 25 °C was performed (Fig. 5). It is seen that an outstanding improvement in uptake of Sr (II) ions is donated using MnO_2_–ZrO_2_-PAN composite. A considerable increment of elimination of (Sr) ions has resulted by substituting amorphous MnO_2_–ZrO_2_ composite with MnO_2_–ZrO_2_-PAN adsorbent.

### The impact of pH

**Figure 6:**
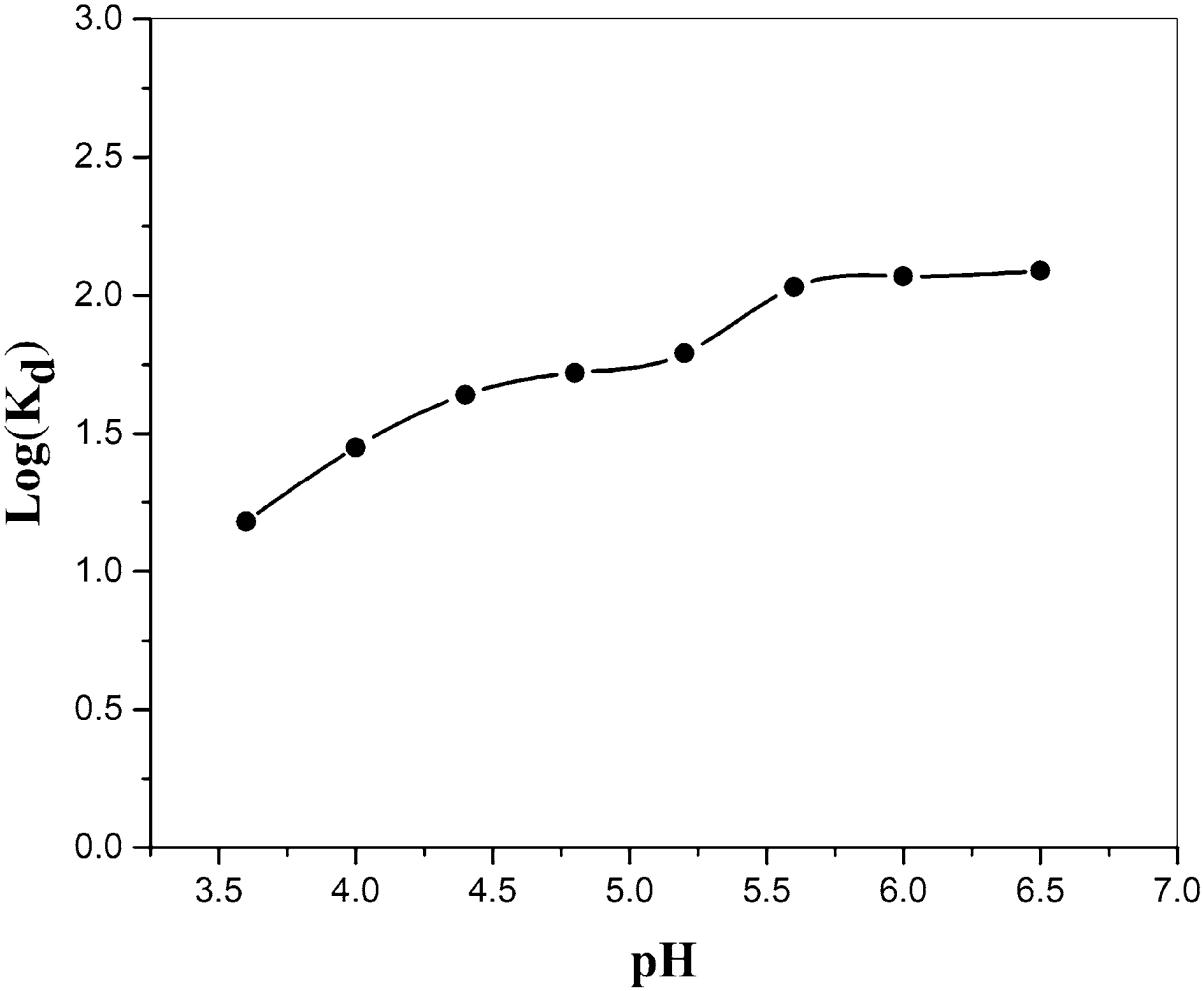
Variation of distribution coefficient of strontium with pH of the solution.

It is figured out that pH is a responsible parameter in the adsorption of Sr^2+^ on MnO_2_–ZrO_2_-PAN composite ion exchangers. To define the optimum situation at which Sr^2+^ are effectually adsorbed on the organized adsorbent, the adsorption runs were performed at various pH ranges from 2.0 to 6.0. 10 ml of 50 mg/L Sr (NO_3_)_2_ mixture was stirred with 0.10 g of MnO_2_–ZrO_2_-PAN for 60 min at 298 K. As could be observed in Fig. 6, the adsorption development continually progressed by improving pH amount from an acidic to an alkali medium. This can be ascribed to the interaction between hydrogen and (Sr) ions for adsorption on the produced composite ion exchangers. It was detected that the highest value of the distribution Coefficient of (Sr) was reached at pH 5.5, and consequently, all adsorption runs in the current work were performed at primary pH 5.5.

According to the obtained results, the adsorption of ions on metal oxide exchangers with a neutral structure is pH dependent, and at higher pH, the surface of the resin becomes negative and the adsorption of metal ions increases. This shows that the attraction of electrostatic interaction between hydrated cations and anionic sites in the exchanger is the reason for the increased adsorption. On the other hand, at low pH, the surface of the resin is protonated and electrostatic repulsion is created between positive metal ions and functional groups of the surface with a positive charge, thus the adsorption decreases^15,21^.

### The impact of shaking time

**Figure 7:**
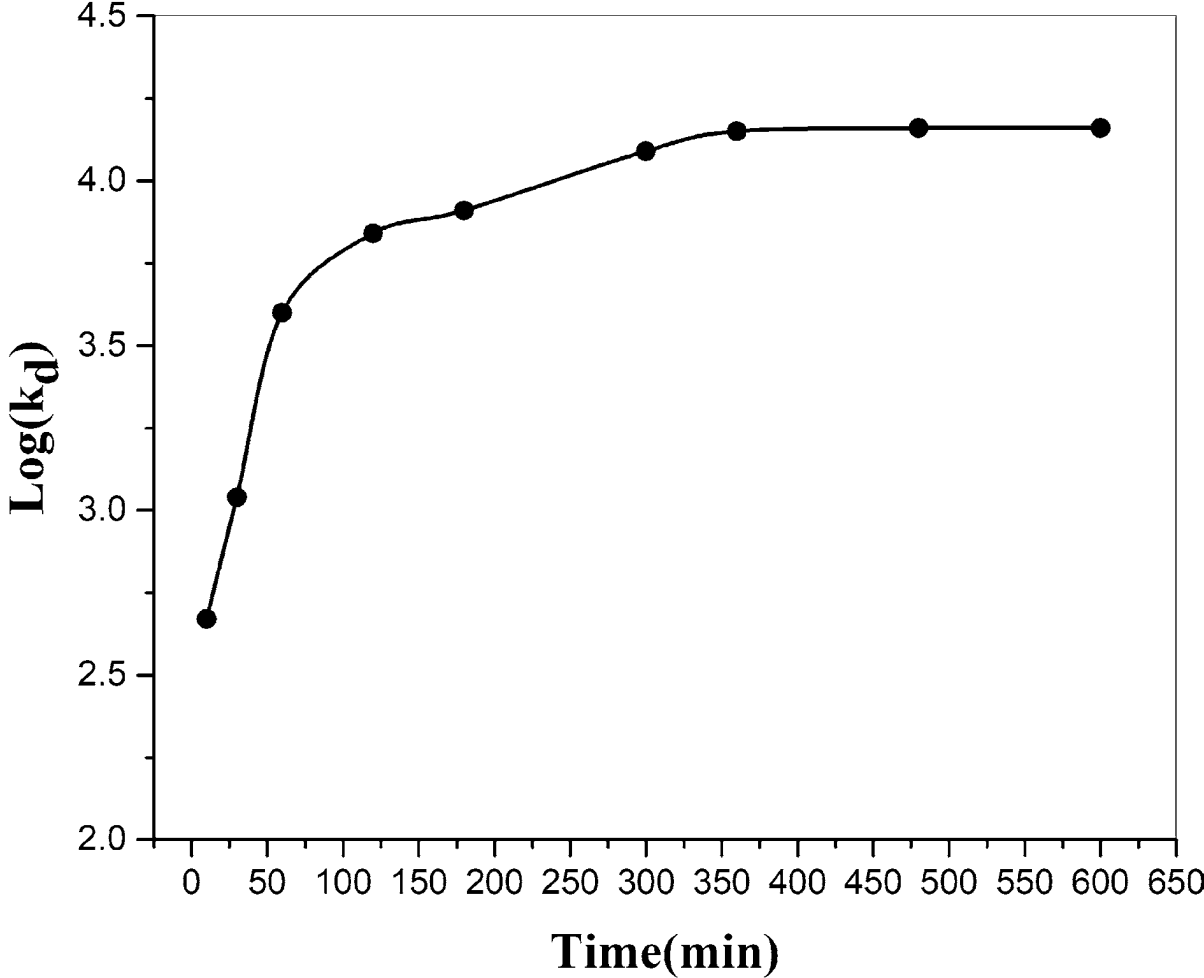
Variation of distribution coefficient of strontium with interaction time.

To verify the impact of interaction time between MnO_2_–ZrO_2_-PAN ion exchangers and solution on Sr^2+^ adsorption, distribution Coefficient (K_d_) changes of Sr^2+^ at pH = 5.5 against time were sketched, as presented in Fig. 7. 10 ml of 50 ppm Sr (NO_3_)_2_ mixture has been stirred with 0.10 g of MnO_2_–ZrO_2_-PAN for various intervals from 10 to 600 min. It was perceived that the adsorption of Sr^2+^ from the mixture through the composite adsorbent is constantly improved with time till achieving an equilibrium state. No significant difference was observed between two phases at longer interaction time and the amount of this leveled off after 6 h.

### Primary ions concentration

**Figure 8:**
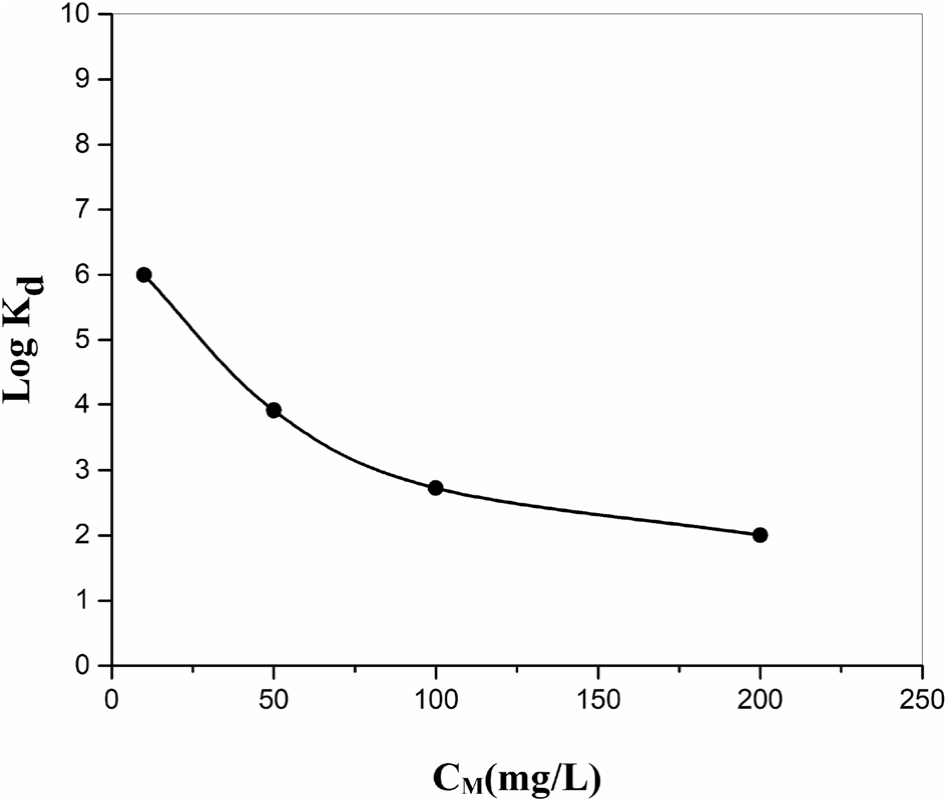
Effect of metal ion concentration on the adsorption of strontium ions onto MnO_2_–ZrO_2_-PAN composite.

To study the value of highest Metal ions adsorbed with a particular quantity of the adsorbent, this parameter was examined by the elimination of the reviewed (MI) with primary concentration in the range 10–200 mg/L by 0.1 g of adsorbent at pH 5.5 (Fig. 8). The amounts of Metal ions adsorbed by MnO_2_–ZrO_2_-PAN reduced regarding the progress of primary concentration of Metal ions. This outcome can be ascribed to the relative reduction in the adsorbing places by intensifying the value of the Metal ions.

### Adsorption kinetic modeling

The kinetic results for Sr^2+^ adsorption from mixtures on the synthesized MnO_2_–ZrO_2_-PAN have been developed by Pseudo-first-order and Pseudo-second-order kinetic models. To examine the precision of these models for estimating the Sr^2+^ adsorption tendency, the correlation coefficient (R^2^) of an individual model, which is a major parameter, was applied. Two general adsorption kinetic models (Pseudo-first-order and Pseudo-second-order correlations) were employed to study the mechanism of adsorption and the impact of interaction time on the adsorption behavior.

The Pseudo-first-order model is illustrated by the subsequent correlation:

4 $$ \ln (q_{e} - q_{t} ) = - k_{1} t + \ln (q_{e} ) $$

where *q*_e_ and *q*_t_ denote the quantities of (Sr)adsorbed on MnO_2_–ZrO_2_-PAN ion exchangers at equilibrium state at specific time *t*, correspondingly (mg/g), and *k*_1_ is the rate constant for the Pseudo-first-order model (min^−1^). The amounts *k*_1_ and *q*_e_ were estimated from the incline and intercept of plot ln (*q*_e_ − *q*_t_) against *t* and were listed in Table 1.

**Figure 9:**
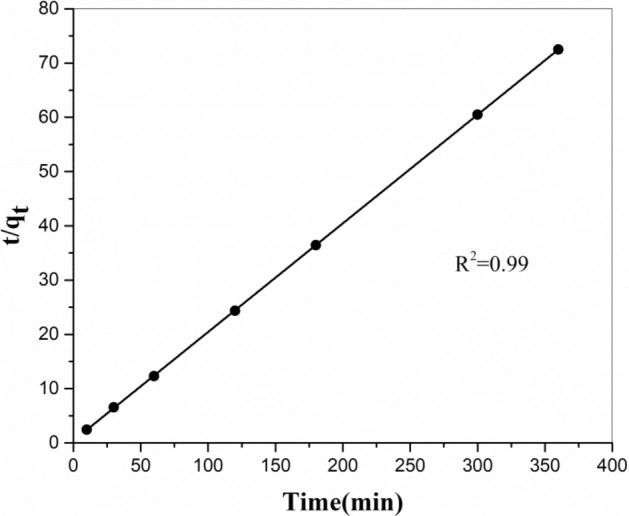
Pseudo-second-order kinetic plot for the sorption of strontium ion onto MnO_2_–ZrO_2_-PAN.

The Pseudo-second-order model may be defined as:

5 $$ \frac{{\text{t}}}{{{\text{q}}_{{\text{t}}} }} = \frac{{1}}{{{\text{k}}_{{2}} {\text{q}}_{{\text{e}}}^{{2}} }} + \frac{{\text{t}}}{{{\text{q}}_{{\text{e}}} }} $$

where *k*_2_ is the rate constant of the Pseudo-second-order equation (g/mg min). The rate constant and equilibrium adsorption capacity were estimated from the incline and intercept of plot *t/q*_t_ against *t* (Fig. 9) and their amounts are mentioned in Table 2. As could be detected, the equation constant for the Pseudo-second-order correlation was greater than that of the Pseudo-first-order correlation, representing that (Sr) adsorption on the produced ion exchangers obeys from the Pseudo-second-order model. These results explain that the Pseudo-second-order adsorption mechanism is predominant and that the overall rate constant of each adsorption process appears to be controlled by the chemical adsorption process^22^. Additionally, it was seen that the estimated *q*_e_ value for the Pseudo-second-order model was compatible with the experimental data. Hence, this model was more appropriate for estimating the kinetic adsorption of (Sr) on MnO_2_–ZrO_2_-PAN composite ion exchangers.

**Table 2:** Parameters of kinetic models for strontium ion sorbent system.

Pseudo-first-order model	
*K*_1_ (min^−1^)	0.023
*q*_e_ (mg/g)	0.771
*R*^*2*^	0.951
Pseudo-second-order model	
*K*_2_ (g/mg min)	0.099
*q*_e_ (mg/g)	5.000
*R*^*2*^	0.99

C_o_, 50 mg/L; *V*, 10 mL; *W*, 0.05 g.

### Adsorption iso-temperature models

Adsorption equilibrium is typically defined by an iso-temperature correlation whose factors explain the surface characteristics and dependence of the adsorbent, at a constant temperature and with a constant pH. An adsorption iso-temperature illustrates the dependence between the value of adsorbate on the adsorbent and the concentration of adsorbate in the solution at an equilibrium state^23^. Many traditional adsorption iso-temperature models involving Langmuir, Freundlich, and Temkin models were taken to fit the achieved iso-temperature results.

#### Langmuir iso-temperature model

**Figure 10:**
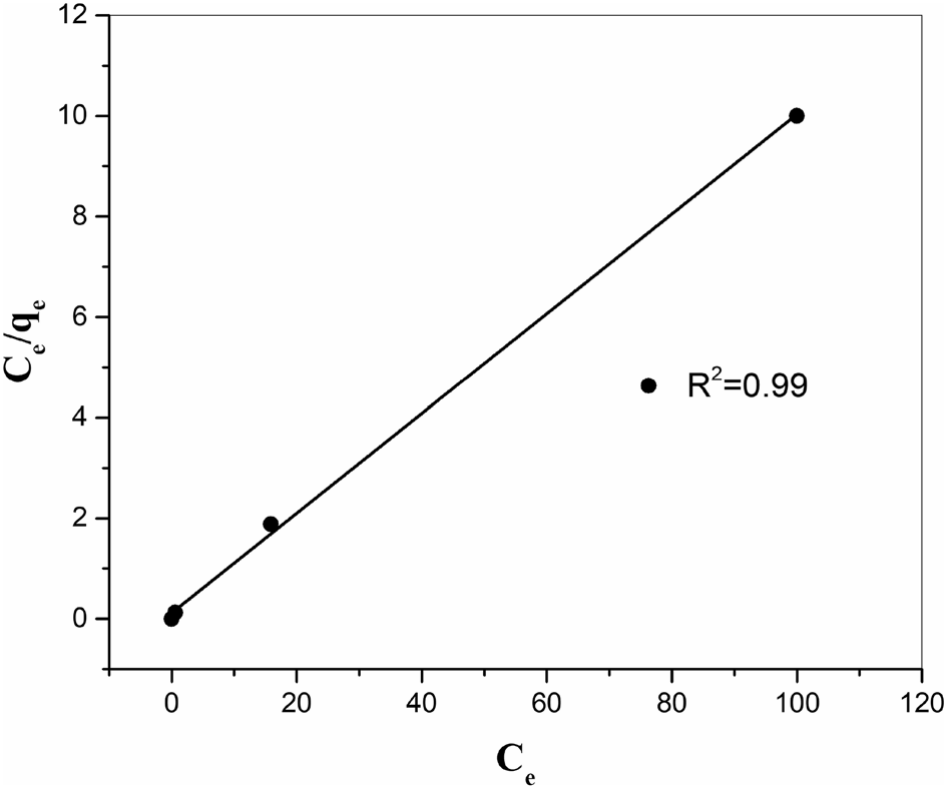
Langmuir adsorption iso-temperature of strontium onto ZrO_2_–MnO_2_-PAN composite at 298 K.

Langmuir adsorption iso-temperature model the monolayer coating of the adsorption sites and suggests that adsorption takes place on a physically homogeneous adsorbent and all the adsorption places are uniform. The linear type of the Langmuir correlation is provided here:

6 $$ \frac{{{\text{C}}_{{\text{e}}} }}{{{\text{q}}_{{\text{e}}} }} = \frac{{{\text{C}}_{{\text{e}}} }}{{{\text{Q}}^{{0}} }} + \frac{{1}}{{{\text{Q}}^{{0}} {\text{b}}}} $$

where *q*_e_ is the value of (MI) adsorbed per unit mass of sorbent (mg/g), *C*_e_ is the equilibrium concentration of the (MI) in the equilibrium solution (mg/L), *Q*^o^ is the monolayer adsorption capacity (mg/g), and *b* is the constant correlated to the adsorption intensity (L/mg). The graphic sketches of (*C*_e_/*q*_e_) versus *C*_e_ give direct lines for Sr^2+^ ions adsorbed onto MnO_2_–ZrO_2_-PAN, as presented in Fig. 10. The mathematical amounts of coefficients *Q*^o^ and *b* estimated from the incline and intercept of the figure are listed in Table 3.

**Table 3:** Parameters of Langmuir, Freundlich and Temkin iso-temperatures for strontium ions sorption onto ZrO_2_–MnO_2_-PAN composite.

Iso-temperatures equations	Parameters	
Langmuir	Q° (mg/g)	10.101
	b (L/mg)	0.839
	R_L_	0.023
	R^2^	0.99
Freundlich	K_f_ (mg/g)	4.487
	n	4.878
	R^2^	0.97
Temkin	a (L/g)	2331.710
	b (kJ/mol)	3.163
	R^2^	0.98

One of the critical properties of the Langmuir method may be explained by dimensionless coefficients termed equilibrium factors *R*_L_^24^.

7 $$ {\text{R}}_{{\text{L}}} = \frac{{1}}{{{1} + {\text{bC}}_{{0}} }} $$

where* C*_0_ is the maximum primary (MI) concentration (mg/L). The amount of *R*_L_ exhibits the kind of iso-temperature to be irreversible (*R*_L_ = 0), satisfactory (0 < *R*_L_ < 1), linear (*R*_L_ = 1), or unsatisfactory (*R*_L_ > 1). The *R*_L_ quantities (Table 3) were figured out to be less than 1 and greater than 0 demonstrating the satisfactory adsorption iso-temperatures of Sr^2+^ ions. The results show that the adsorption of strontium occurs at a single layer on the resin surface that has a homogeneous structure, and all adsorption sites are the same in terms of energy^22^.

#### Freundlich iso-temperature model

**Figure 11:**
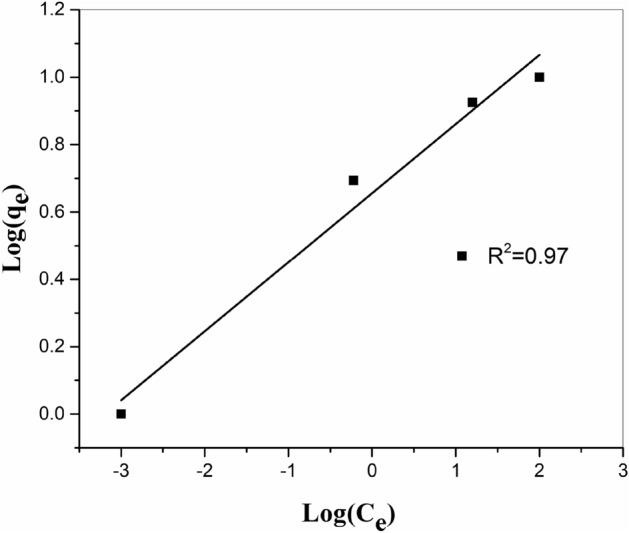
Freundlich adsorption iso-temperature of strontium onto ZrO_2_–MnO_2_-PAN composite at 298 K.

Freundlich correlation originated to model the multilayer adsorption and the adsorption on nonhomogeneous sites. The logarithmic type of Freundlich correlation can be explained as:

8 $$ {\text{Logq}}_{{\text{e}}} = {\text{LogK}}_{{\text{f}}} + \frac{{1}}{{\text{n}}}{\text{LogC}}_{{\text{e}}} $$

where *K*_f_ is the constant as an indicator of the relative adsorption capacity of MnO_2_–ZrO_2_-PAN (mg/g) and 1/*n* is the constant which indicate the severity of the adsorption behavior. The pictorial imagination of log_ge_d against log*C*_e_ is seen in Fig. 11. The mathematical amounts of the constants 1/*n* and *K*_f_ are calculated from the incline and the intercepts, through a linear least square fitting model, which one written in Table3.

In this case, *n* > 1 for Sr^2+^, the MnO_2_–ZrO_2_-PAN exhibits an improving propensity for adsorption with enhancing solid phase concentration. This can be ascribed to the fact that with the developing surface coating of the adsorbent, the attractive forces among the (MI) elements such as van der-Waals forces, improve more quickly than the repulsive forces, demonstrated by short-range electronic or long-range Coulombic dipole repulsion, and subsequently, the Metal ions disclose a stronger affinity to bind to the MnO_2_–ZrO_2_-PAN site^25,26^.

#### Temkin iso-temperature

**Figure 12:**
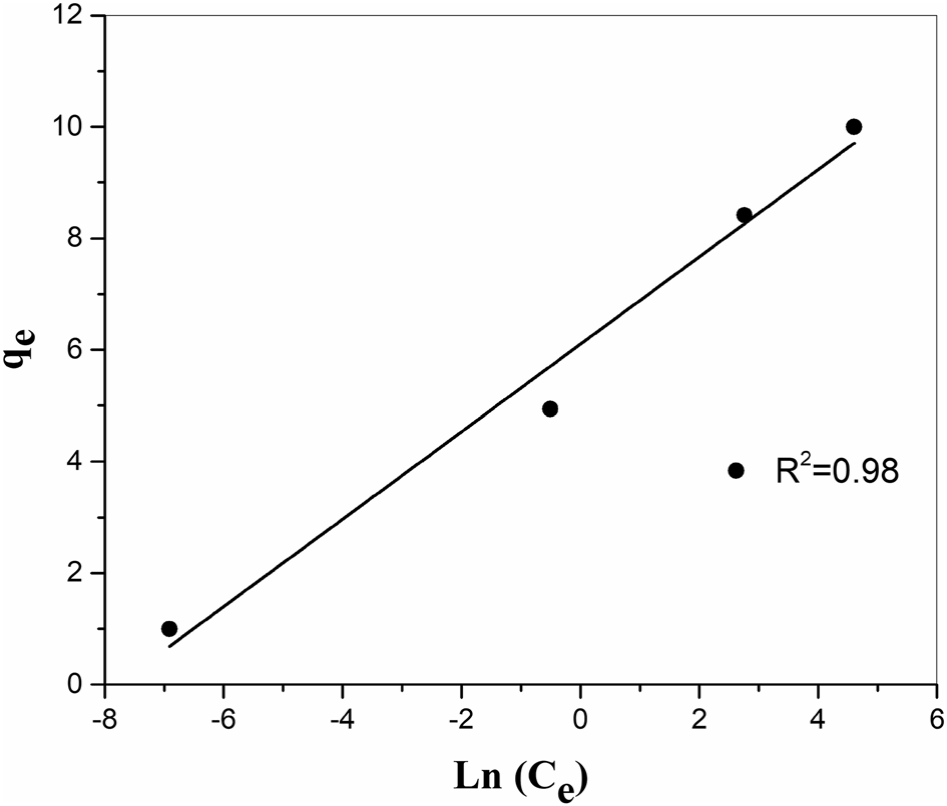
Temkin adsorption iso-temperature of strontium onto ZrO_2_–MnO_2_-PAN composite at 298 K.

Temkin iso-temperature correlation that studies the impacts of the heat of adsorption which reduces linearly with coating of the adsorbate and adsorbent interactions, was utilized in the linear type as bellows^27^:

9 $$ {\text{q}}_{{\text{e}}} = \frac{{{\text{RT}}}}{{\text{b}}}{\text{ln(aC}}_{{\text{e}}} {)} $$

where b is the Temkin constant regarding heat of adsorption (kJ/mol) and a is the Temkin iso-temperature coefficient (L/g). Sketching q_e_ against ln(C_e_) is seen in Fig. 12 allowing estimating a, b, and the obtained (R^2^) of the result (Table 3).

Ultimately, it should be mentioned that the usability of all the iso-temperature models studied to the (Sr) adsorption process from waste stream displays that both monolayer adsorption and nonhomogeneous energetic dispersion of active parts on the adsorbent surface are feasible^28^. From the obtained (R^2^) quantities for those models in Table 3, it was obvious that the iso-temperature models usability for (Sr)extraction via the synthesized MnO_2_–ZrO_2_-PAN composite obeys from the order: Langmuir > Frendlich > Temkin iso-temperature. Additionally, the estimated R_L_ (0.023), in the Langmuir model, proves the satisfactory uptake of (Sr)ions by the tested adsorbent.

### Adsorption analysis

To define the potential capability of the ZrO_2_–MnO_2_-PAN composite in the removal of Metal ions, distribution Coefficient (K_d_) investigations for various Metal ions in the optimum situations were performed and relative standard deviations (RSDs) were calculated. The data are presented in Table 4. The distribution Coefficient of the Metal ions on this adsorbent revealed a high dependence of this substance for some ions for instance, Sr (II), Y(III), Ni (II), Pb (II), and Co (II) in water. The distribution of the Sr (II), Y(III), Ni (II), Pb (II), and Co (II) ions against others was defined by the proportion of the two distribution Coefficient, K_d_M_2_ and K_d_M_1_, that is called the selectivity factor. In order to evaluate the adsorption selectivity of adsorbents, the separation factor is defined as follows:

**Table 4:** Distribution coefficients of metal ions onto of MnO_2_–ZrO_2_-PAN and relative standard deviations (RSDs).

Metal ions	K_d_ values (mL/g)	%RSD
Sr (II)	*T.A	N/A
Zr (IV)	468	1.92
Mo (VI)	365	2.34
Pb (II)	8533	1.15
Co (II)	6200	1.45
Y (III)	T.A	N/A
Hg (II)	870	2.29
Ni (II)	T.A	N/A
La (III)	12	4.16

*Total Adsorption (~ 10E6).

10 $$\propto =\frac{{K}_{{d}_{{M}_{2}}}}{{K}_{{d}_{{M}_{1}}}}$$

The attained data are briefed in Table 5. It is evident from the data that the quantifiable removal of (Sr) ions from Zr (IV), Mo (VI), and La (III) is feasible. The results revealed that the adsorption efficiency of strontium for the resin synthesized in this work is higher compared to other adsorbents reported such as PAN-Zeolite, Natural Clinoptilolite, Natural Attapulgite and Ca-alginate^29^.

**Table 5:** Selectivity coefficients of Sr(II), Y(III), Ni(II), Pb(II) and Co(II) ions over other inorganic cations.

Metal ions	Selectivity factor (α)		
	For Co(II)	For Pb(II)	For Sr(II), Ni(II), Y(III)
Zr (IV)	13.25	18.23	> 1000
Mo (VI)	16.99	23.38	> 1000
Hg (II)	7.13	9.81	> 1000
La (III)	516.67	711.08	> 10^5^

### Desorption analysis

**Figure 13:**
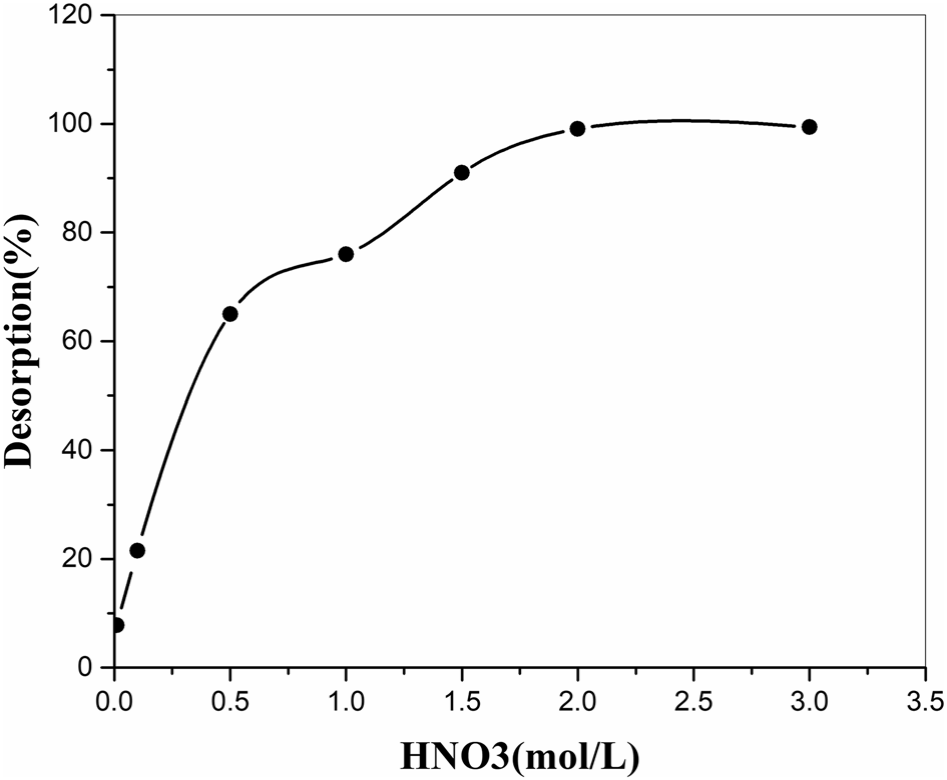
Effect of Nitric concentration on the desorption of strontium ions onto MnO_2_–ZrO_2_-PAN composite.

To investigate the desorption of strontium ions, 0.1 g of the resin on which the maximum strontium was absorbed was shaken with 10 ml of Nitric acid solution (0.01–3M). As shown in Fig. 13, an effective result was obtained with 2M HNO_3_, and the desorption efficiency obtained was roughly 99%.

## Conclusion

Modified inorganic ion exchangers with polyacrylonitrile MnO_2_–ZrO_2_-PAN was figured out to have useful ion exchanger capacity and thermal stability and high selectivity for strontium. The adsorption characteristics exhibited that the adsorption of (Sr) is entirely dependent on the interaction time, primary concentration ion, and pH of the media employed, and as these controlling factors grow, the adsorption of (Sr) ions for the ion exchangers improves, at least for a pH up to 6. The results showed that the adsorption of strontium on MnO_2_–ZrO_2_-PAN is pH dependent, and at high pH, the surface of the resin becomes negative and the adsorption of metal ions increases. The kinetics of strontium adsorption was in good agreement with the Pseudo second-order model and demonstrated that the adsorption rate constant controlled by the chemical adsorption process. The Langmuir model method can be considered to be the best among other models; therefore, strontium adsorption occurs at a single layer on the surface of the resin. It also revealed a powerful dependence regarding the Y (III), Ni (II), Pb (II), and Co (II). Evaluation of strontium desorption showed that the maximum strontium desorption was at 2 M nitric acid. Consequently, the produced composite may be employed to reject and isolate those heavy poisonous metals from the nuclear waste streams.

## Data Availability

The datasets used and/or analyzed during the current study are available from the corresponding author on reasonable request.
